# (20*S*)-24,25-Dihydr­oxy-20,24-ep­oxy-3,4-secodammar-4(28)-en-3-oic acid from *Aglaia smithii*

**DOI:** 10.1107/S1600536810002072

**Published:** 2010-01-23

**Authors:** Desi Harneti, Unang Supratman, Mat Ropi Mukhtar, Khalijah Awang, Seik Weng Ng

**Affiliations:** a Department of Chemistry, Faculty of Mathematics and Natural Sciences, Padjadjaran University, Jatinangor 45363, West Java, Indonesia; b Department of Chemistry, University of Malaya, 50603 Kuala Lumpur, Malaysia

## Abstract

The title compound, C_30_H_50_O_5_, was isolated from the bark of *Aglaia smithii*. There are two independent mol­ecules in the asymmetric unit that differ in the orientation of the isopropenyl group attached to the cyclo­hexane ring. The cyclo­hexane rings in both mol­ecules adopt chair conformations, whereas the cyclo­pentane and tetra­hydro­furan rings adopt envelope conformations. The independent mol­ecules are linked into a layer parallel to (010) by O—H⋯O hydrogen bonds.

## Related literature

For the spectroscopic characterization of 24-ep­oxy-24,25-dihydr­oxy-3,4-secodammar-4(28)-en-3-oic acid from different plants, see: de Campos Braga *et al.* (2006 ▶); Luo *et al.* (2000 ▶); Mohamad *et al.* (1999 ▶).
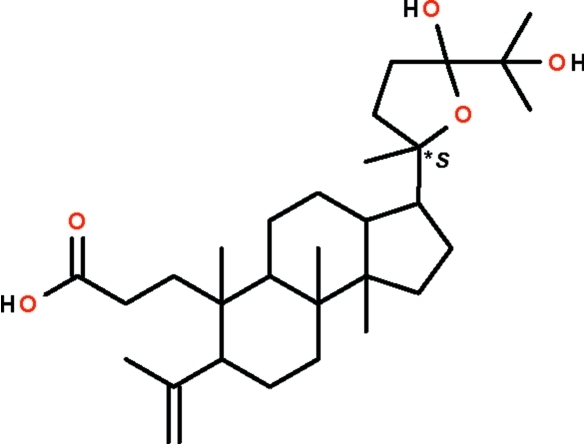


## Experimental

### 

#### Crystal data

C_30_H_50_O_5_
*M* *_r_* = 490.70
Orthorhombic, 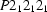

*a* = 7.7322 (1) Å
*b* = 22.4976 (4) Å
*c* = 31.6043 (5) Å
*V* = 5497.76 (15) Å^3^
*Z* = 8
Mo *K*α radiation
μ = 0.08 mm^−1^
*T* = 123 K
0.45 × 0.15 × 0.10 mm

#### Data collection

Bruker SMART APEX diffractometer
52832 measured reflections
7026 independent reflections
5590 reflections with *I* > 2σ(*I*)
*R*_int_ = 0.058

#### Refinement

*R*[*F* ^2^ > 2σ(*F* ^2^)] = 0.064
*wR*(*F* ^2^) = 0.175
*S* = 1.09
7026 reflections
651 parameters
H-atom parameters constrained
Δρ_max_ = 0.64 e Å^−3^
Δρ_min_ = −0.26 e Å^−3^

### 

Data collection: *APEX2* (Bruker, 2008 ▶); cell refinement: *SAINT* (Bruker, 2008 ▶); data reduction: *SAINT*; program(s) used to solve structure: *SHELXS97* (Sheldrick, 2008 ▶); program(s) used to refine structure: *SHELXL97* (Sheldrick, 2008 ▶); molecular graphics: *X-SEED* (Barbour, 2001 ▶); software used to prepare material for publication: *publCIF* (Westrip, 2010 ▶).

## Supplementary Material

Crystal structure: contains datablocks global, I. DOI: 10.1107/S1600536810002072/ci5019sup1.cif

Structure factors: contains datablocks I. DOI: 10.1107/S1600536810002072/ci5019Isup2.hkl

Additional supplementary materials:  crystallographic information; 3D view; checkCIF report

## Figures and Tables

**Table 1:** Hydrogen-bond geometry (Å, °)

*D*—H⋯*A*	*D*—H	H⋯*A*	*D*⋯*A*	*D*—H⋯*A*
O1—H1⋯O10	0.84	1.91	2.709 (4)	158
O4—H4⋯O7^i^	0.84	1.96	2.770 (4)	160
O5—H5⋯O7^i^	0.84	2.45	3.205 (5)	149
O6—H6⋯O5^ii^	0.84	1.99	2.780 (5)	157
O9—H9⋯O2	0.84	1.98	2.807 (5)	171
O10—H10⋯O5^iii^	0.84	2.15	2.829 (4)	138

Symmetry codes: (i) 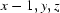; (ii) 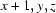; (iii) 
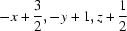
.

**Fig. 1.**
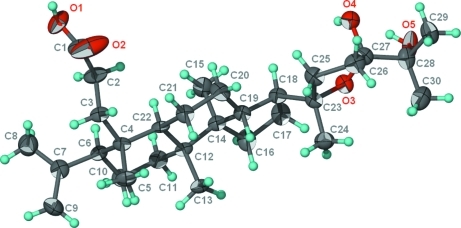
Displacement ellipsoid plot (Barbour, 2001) of one of the two independent molecules in the title compound, at the 70% probability level. H atoms are drawn as spheres of arbitrary radius.

**Fig. 2.**
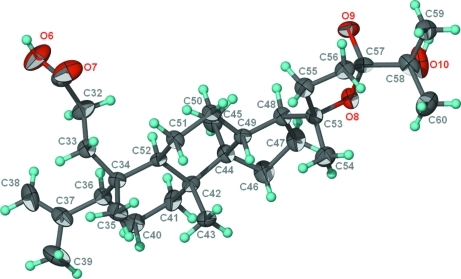
Displacement ellipsoid plot (Barbour, 2001) of second independent molecule in the title compound, at the 70% probability level. H atoms are drawn as spheres of arbitrary radius.

## supplementary crystallographic information

### Comment

*Aglaia* is a genus of more than 100 species belonging to the Mahagony family (Meliaceae). The trees are found in the tropical and subtropical forests of Southeast Asia, Northern Australia and the Pacific. Some are commercially important timber trees; others yield edible fruit and scented flowers whereas others are used for medicinal purposes.

### Experimental

*Aglaia smithii* was collected in the Bogor Botanical Garden, West Java, Indonesia; the plant was identified by the Herbarium Bogoriense in Bogor. The dried and milled bark of *A. smithii* (5 kg) was extracted exhaustively by methanol at room temperature to yield a concentrated methanol extract (0.7 kg); 300 g of the extract was subjected to vacuum column chromatography on silica gel 60 by using a step gradient of *n*-hexane–ethylacetate–methanol. The fraction eluted by *n*-hexane–ethylacetate (1:1) was further separated by column chromatography on silica gel (chloroform: methanol 9.75:0.25) to give 20*S*,24-epoxy-24,25-dihydroxy-3,4-secodammar-4(28)-en-3-oic acid (34 mg). The structure was established by ^1^H-NMR spectroscopic analysis. The IR spectrum showed the carbonyl stretching at 1710 cm^-1^.

### Refinement

H-atoms were placed in calculated positions (O-H = 0.84 Å and C-H = 0.95–1.00 Å) and were included in the refinement in the riding model approximation, with *U_iso_*(H) = 1.2*U_eq_*(C) and 1.5*U_eq_*(O,C_methyl_). A rotating group model was used for the –OH and –CH_3_ groups. The structure features a short H5···H6 (*x*-1,*y*,*z*) contact of 1.83 Å.

In the absence of significant anomalous dispersion effects, Friedel pairs were merged before the final refinement. Therefore, the absolute configuration reported in the literature (de Campos Braga *et al.*, 2006; Luo *et al.*, 2000; Mohamad *et al.*, 1999) has been assigned.

### Crystal data

**Table:**

C_30_H_50_O_5_	*F*(000) = 2160
*M**_r_* = 490.70	*D*_x_ = 1.186 Mg m^−^^3^
Orthorhombic, *P*2_1_2_1_2_1_	Mo *K*α radiation, λ = 0.71073 Å
Hall symbol: P 2ac 2ab	Cell parameters from 8963 reflections
*a* = 7.7322 (1) Å	θ = 2.6–27.6°
*b* = 22.4976 (4) Å	µ = 0.08 mm^−^^1^
*c* = 31.6043 (5) Å	*T* = 123 K
*V* = 5497.76 (15) Å^3^	Block, colourless
*Z* = 8	0.45 × 0.15 × 0.10 mm

### Data collection

**Table:**

Bruker SMART APEX diffractometer	5590 reflections with *I* > 2σ(*I*)
Radiation source: fine-focus sealed tube	*R*_int_ = 0.058
graphite	θ_max_ = 27.5°, θ_min_ = 1.1°
ω scans	*h* = −9→10
52832 measured reflections	*k* = −28→29
7026 independent reflections	*l* = −41→41

### Refinement

**Table:**

Refinement on *F*^2^	Primary atom site location: structure-invariant direct methods
Least-squares matrix: full	Secondary atom site location: difference Fourier map
*R*[*F*^2^ > 2σ(*F*^2^)] = 0.064	Hydrogen site location: inferred from neighbouring sites
*wR*(*F*^2^) = 0.175	H-atom parameters constrained
*S* = 1.09	*w* = 1/[σ^2^(*F*_o_^2^) + (0.0818*P*)^2^ + 3.9232*P*] where *P* = (*F*_o_^2^ + 2*F*_c_^2^)/3
7026 reflections	(Δ/σ)_max_ = 0.001
651 parameters	Δρ_max_ = 0.64 e Å^−^^3^
0 restraints	Δρ_min_ = −0.25 e Å^−^^3^

### Fractional atomic coordinates and isotropic or equivalent isotropic displacement parameters (Å^2^)

**Table:**

	*x*	*y*	*z*	*U*_iso_*/*U*_eq_	
O1	0.7294 (4)	0.59393 (14)	0.80831 (11)	0.0442 (8)	
H1	0.8240	0.6111	0.8128	0.066*	
O2	0.9021 (5)	0.51630 (17)	0.79778 (18)	0.0762 (14)	
O3	0.5230 (3)	0.36487 (13)	0.51958 (8)	0.0286 (6)	
O4	0.6886 (4)	0.44300 (12)	0.49404 (9)	0.0321 (6)	
H4	0.5922	0.4592	0.4982	0.048*	
O5	0.5052 (4)	0.39226 (15)	0.43025 (9)	0.0431 (8)	
H5	0.5062	0.4267	0.4405	0.065*	
O6	1.1987 (4)	0.42773 (16)	0.46868 (13)	0.0564 (10)	
H6	1.2929	0.4109	0.4631	0.085*	
O7	1.3750 (4)	0.50166 (15)	0.48684 (13)	0.0498 (9)	
O8	1.0443 (3)	0.65801 (13)	0.75888 (8)	0.0287 (6)	
O9	1.2105 (4)	0.57900 (12)	0.78355 (10)	0.0329 (6)	
H9	1.1125	0.5634	0.7864	0.049*	
O10	1.0195 (4)	0.63938 (18)	0.84383 (9)	0.0468 (9)	
H10	1.0447	0.6161	0.8637	0.070*	
C1	0.7578 (6)	0.5385 (2)	0.79858 (16)	0.0397 (11)	
C2	0.5988 (5)	0.50533 (19)	0.78545 (15)	0.0360 (10)	
H2A	0.5878	0.5071	0.7543	0.043*	
H2B	0.4960	0.5250	0.7978	0.043*	
C3	0.6021 (5)	0.44070 (16)	0.79936 (13)	0.0259 (8)	
H3A	0.5926	0.4397	0.8306	0.031*	
H3B	0.7166	0.4240	0.7919	0.031*	
C4	0.4605 (5)	0.39870 (16)	0.78083 (12)	0.0233 (8)	
C5	0.5108 (5)	0.33588 (16)	0.79598 (13)	0.0298 (9)	
H5A	0.5372	0.3370	0.8263	0.045*	
H5B	0.6128	0.3222	0.7803	0.045*	
H5C	0.4144	0.3085	0.7909	0.045*	
C6	0.2795 (5)	0.41845 (16)	0.79768 (12)	0.0243 (8)	
H6A	0.2658	0.4610	0.7891	0.029*	
C7	0.2572 (5)	0.41713 (19)	0.84555 (13)	0.0312 (9)	
C8	0.2569 (7)	0.4730 (2)	0.86775 (15)	0.0464 (12)	
H8A	0.2266	0.4663	0.8975	0.070*	
H8B	0.1718	0.4997	0.8548	0.070*	
H8C	0.3720	0.4910	0.8661	0.070*	
C9	0.2250 (7)	0.3648 (2)	0.86775 (15)	0.0442 (11)	
H9A	0.2030	0.3662	0.8973	0.053*	
H9B	0.2250	0.3278	0.8533	0.053*	
C10	0.1322 (5)	0.38449 (19)	0.77585 (13)	0.0300 (9)	
H10A	0.1432	0.3416	0.7823	0.036*	
H10B	0.0200	0.3984	0.7873	0.036*	
C11	0.1343 (5)	0.39333 (19)	0.72812 (13)	0.0282 (8)	
H11A	0.1119	0.4357	0.7218	0.034*	
H11B	0.0393	0.3698	0.7154	0.034*	
C12	0.3060 (4)	0.37500 (15)	0.70726 (12)	0.0213 (7)	
C13	0.3180 (5)	0.30636 (16)	0.70847 (13)	0.0283 (8)	
H13A	0.2470	0.2896	0.6857	0.042*	
H13B	0.2760	0.2919	0.7358	0.042*	
H13C	0.4386	0.2942	0.7046	0.042*	
C14	0.3139 (5)	0.39729 (16)	0.66023 (12)	0.0244 (8)	
C15	0.2875 (6)	0.46553 (18)	0.65651 (14)	0.0356 (9)	
H15A	0.3691	0.4860	0.6753	0.053*	
H15B	0.1689	0.4757	0.6647	0.053*	
H15C	0.3079	0.4780	0.6272	0.053*	
C16	0.1854 (5)	0.3694 (2)	0.62816 (13)	0.0342 (9)	
H16A	0.1747	0.3261	0.6327	0.041*	
H16B	0.0696	0.3879	0.6307	0.041*	
C17	0.2658 (5)	0.3828 (2)	0.58458 (13)	0.0358 (10)	
H17A	0.2169	0.4199	0.5727	0.043*	
H17B	0.2426	0.3498	0.5646	0.043*	
C18	0.4646 (5)	0.38968 (18)	0.59222 (12)	0.0265 (8)	
H18	0.4963	0.4320	0.5864	0.032*	
C19	0.4877 (5)	0.37863 (17)	0.63995 (11)	0.0240 (8)	
H19	0.4984	0.3347	0.6438	0.029*	
C20	0.6392 (5)	0.4069 (2)	0.66317 (13)	0.0341 (10)	
H20A	0.6351	0.4506	0.6595	0.041*	
H20B	0.7491	0.3923	0.6509	0.041*	
C21	0.6333 (5)	0.39158 (19)	0.71061 (12)	0.0277 (8)	
H21A	0.6595	0.3488	0.7142	0.033*	
H21B	0.7245	0.4144	0.7254	0.033*	
C22	0.4582 (4)	0.40510 (16)	0.73132 (12)	0.0215 (7)	
H22	0.4407	0.4486	0.7264	0.026*	
C23	0.5735 (5)	0.35016 (18)	0.56319 (12)	0.0267 (8)	
C24	0.5401 (6)	0.28394 (18)	0.56902 (14)	0.0360 (9)	
H24A	0.4166	0.2758	0.5651	0.054*	
H24B	0.5749	0.2720	0.5976	0.054*	
H24C	0.6072	0.2615	0.5481	0.054*	
C25	0.7685 (5)	0.3640 (2)	0.56435 (13)	0.0313 (9)	
H25A	0.7898	0.4052	0.5740	0.038*	
H25B	0.8303	0.3362	0.5833	0.038*	
C26	0.8245 (5)	0.3557 (2)	0.51893 (13)	0.0331 (9)	
H26A	0.8439	0.3132	0.5122	0.040*	
H26B	0.9309	0.3785	0.5126	0.040*	
C27	0.6690 (5)	0.38041 (18)	0.49500 (12)	0.0279 (8)	
C28	0.6429 (5)	0.3572 (2)	0.44935 (13)	0.0324 (9)	
C29	0.8056 (6)	0.3656 (2)	0.42355 (13)	0.0385 (10)	
H29A	0.7845	0.3530	0.3943	0.058*	
H29B	0.8991	0.3417	0.4357	0.058*	
H29C	0.8388	0.4076	0.4239	0.058*	
C30	0.5795 (7)	0.2937 (2)	0.44868 (17)	0.0481 (12)	
H30A	0.5898	0.2777	0.4199	0.072*	
H30B	0.4581	0.2924	0.4576	0.072*	
H30C	0.6494	0.2697	0.4681	0.072*	
C31	1.2290 (5)	0.48154 (19)	0.48244 (14)	0.0343 (9)	
C32	1.0690 (5)	0.51515 (19)	0.49387 (15)	0.0352 (10)	
H32A	1.0532	0.5140	0.5249	0.042*	
H32B	0.9677	0.4956	0.4807	0.042*	
C33	1.0769 (5)	0.57956 (18)	0.47940 (12)	0.0265 (8)	
H33A	1.0621	0.5802	0.4483	0.032*	
H33B	1.1944	0.5948	0.4855	0.032*	
C34	0.9440 (5)	0.62359 (16)	0.49894 (12)	0.0230 (8)	
C35	1.0015 (6)	0.68495 (18)	0.48339 (13)	0.0322 (9)	
H35A	1.0992	0.6988	0.5005	0.048*	
H35B	0.9052	0.7130	0.4860	0.048*	
H35C	1.0370	0.6823	0.4537	0.048*	
C36	0.7585 (5)	0.60654 (18)	0.48330 (12)	0.0267 (8)	
H36	0.7412	0.5642	0.4918	0.032*	
C37	0.7305 (5)	0.6083 (2)	0.43542 (13)	0.0349 (10)	
C38	0.7119 (7)	0.5543 (2)	0.41364 (14)	0.0483 (12)	
H38A	0.6871	0.5543	0.3842	0.058*	
H38B	0.7241	0.5177	0.4283	0.058*	
C39	0.7118 (7)	0.6628 (2)	0.41294 (16)	0.0533 (13)	
H39A	0.6750	0.6545	0.3839	0.080*	
H39B	0.8228	0.6838	0.4125	0.080*	
H39C	0.6247	0.6875	0.4270	0.080*	
C40	0.6166 (5)	0.6419 (2)	0.50618 (13)	0.0332 (9)	
H40A	0.6317	0.6848	0.5003	0.040*	
H40B	0.5019	0.6297	0.4953	0.040*	
C41	0.6240 (5)	0.6313 (2)	0.55383 (13)	0.0313 (9)	
H41A	0.6017	0.5887	0.5595	0.038*	
H41B	0.5310	0.6545	0.5676	0.038*	
C42	0.7977 (4)	0.64850 (16)	0.57358 (12)	0.0234 (7)	
C43	0.8121 (6)	0.71613 (18)	0.57232 (13)	0.0348 (9)	
H43A	0.8005	0.7299	0.5430	0.052*	
H43B	0.9249	0.7283	0.5835	0.052*	
H43C	0.7200	0.7336	0.5896	0.052*	
C44	0.8111 (5)	0.62521 (17)	0.62067 (11)	0.0246 (8)	
C45	0.7762 (6)	0.55771 (18)	0.62425 (13)	0.0374 (10)	
H45A	0.8167	0.5434	0.6518	0.056*	
H45B	0.8381	0.5368	0.6017	0.056*	
H45C	0.6519	0.5502	0.6216	0.056*	
C46	0.6928 (5)	0.6556 (2)	0.65346 (13)	0.0367 (10)	
H46A	0.6904	0.6992	0.6492	0.044*	
H46B	0.5732	0.6401	0.6516	0.044*	
C47	0.7754 (5)	0.6398 (2)	0.69631 (13)	0.0337 (9)	
H48A	0.7241	0.6028	0.7078	0.040*	
H48B	0.7569	0.6723	0.7170	0.040*	
C48	0.9731 (5)	0.63108 (18)	0.68749 (12)	0.0258 (8)	
H48	1.0034	0.5888	0.6937	0.031*	
C49	0.9898 (4)	0.64121 (16)	0.63916 (11)	0.0229 (7)	
H49	1.0044	0.6850	0.6351	0.027*	
C50	1.1350 (5)	0.61177 (19)	0.61497 (12)	0.0281 (8)	
H50A	1.1259	0.5680	0.6177	0.034*	
H50B	1.2478	0.6243	0.6268	0.034*	
C51	1.1247 (4)	0.62944 (18)	0.56807 (12)	0.0254 (8)	
H51A	1.1515	0.6723	0.5653	0.030*	
H51B	1.2135	0.6071	0.5521	0.030*	
C52	0.9462 (4)	0.61729 (16)	0.54841 (11)	0.0200 (7)	
H52	0.9265	0.5739	0.5535	0.024*	
C53	1.0877 (5)	0.67116 (17)	0.71490 (12)	0.0250 (8)	
C54	1.0552 (6)	0.73665 (18)	0.70817 (14)	0.0361 (10)	
H54A	1.1175	0.7595	0.7297	0.054*	
H54B	0.9310	0.7447	0.7104	0.054*	
H54C	1.0961	0.7482	0.6800	0.054*	
C55	1.2821 (5)	0.65641 (19)	0.71187 (13)	0.0295 (8)	
H55A	1.3002	0.6149	0.7026	0.035*	
H55B	1.3415	0.6835	0.6919	0.035*	
C56	1.3465 (5)	0.66561 (19)	0.75689 (13)	0.0307 (9)	
H56A	1.3673	0.7082	0.7630	0.037*	
H56B	1.4537	0.6428	0.7624	0.037*	
C57	1.1936 (5)	0.64126 (17)	0.78243 (12)	0.0272 (8)	
C58	1.1739 (5)	0.6655 (2)	0.82744 (13)	0.0328 (9)	
C59	1.3286 (6)	0.6499 (2)	0.85434 (14)	0.0377 (10)	
H59A	1.3099	0.6642	0.8833	0.057*	
H59B	1.4322	0.6688	0.8426	0.057*	
H59C	1.3442	0.6067	0.8547	0.057*	
C60	1.1432 (7)	0.7328 (2)	0.82780 (17)	0.0512 (13)	
H60A	1.1197	0.7460	0.8568	0.077*	
H60B	1.0440	0.7424	0.8097	0.077*	
H60C	1.2463	0.7532	0.8171	0.077*	

### Atomic displacement parameters (Å^2^)

**Table:**

	*U*^11^	*U*^22^	*U*^33^	*U*^12^	*U*^13^	*U*^23^
O1	0.0224 (14)	0.0460 (19)	0.064 (2)	−0.0072 (13)	0.0018 (15)	−0.0262 (16)
O2	0.0296 (19)	0.038 (2)	0.161 (5)	−0.0036 (16)	−0.015 (2)	−0.004 (2)
O3	0.0162 (12)	0.0399 (16)	0.0296 (14)	0.0005 (12)	−0.0025 (11)	0.0011 (12)
O4	0.0264 (14)	0.0343 (15)	0.0357 (15)	0.0027 (12)	−0.0001 (13)	0.0000 (12)
O5	0.0375 (17)	0.057 (2)	0.0344 (16)	0.0252 (16)	−0.0121 (14)	−0.0182 (15)
O6	0.0272 (16)	0.055 (2)	0.087 (3)	0.0113 (16)	−0.0014 (18)	−0.0274 (19)
O7	0.0229 (15)	0.044 (2)	0.083 (3)	0.0030 (14)	0.0006 (16)	−0.0024 (18)
O8	0.0155 (12)	0.0454 (17)	0.0251 (13)	−0.0011 (12)	0.0031 (10)	−0.0009 (12)
O9	0.0328 (15)	0.0230 (14)	0.0429 (16)	0.0003 (12)	−0.0072 (14)	0.0010 (12)
O10	0.0361 (17)	0.077 (3)	0.0274 (15)	−0.0305 (18)	0.0053 (13)	−0.0096 (15)
C1	0.026 (2)	0.034 (2)	0.059 (3)	−0.0063 (18)	−0.013 (2)	0.007 (2)
C2	0.025 (2)	0.031 (2)	0.052 (3)	−0.0075 (17)	−0.0110 (19)	0.0033 (19)
C3	0.0172 (17)	0.0254 (19)	0.035 (2)	−0.0022 (14)	−0.0071 (15)	0.0023 (16)
C4	0.0184 (16)	0.0224 (18)	0.0290 (19)	−0.0026 (14)	−0.0040 (14)	0.0052 (15)
C5	0.0274 (19)	0.0218 (19)	0.040 (2)	0.0037 (16)	−0.0041 (17)	0.0077 (16)
C6	0.0204 (17)	0.0210 (18)	0.0315 (19)	0.0002 (14)	−0.0013 (15)	−0.0008 (15)
C7	0.0199 (18)	0.040 (2)	0.034 (2)	−0.0004 (17)	−0.0030 (16)	−0.0002 (17)
C8	0.057 (3)	0.044 (3)	0.038 (2)	0.004 (2)	−0.003 (2)	0.000 (2)
C9	0.051 (3)	0.045 (3)	0.037 (2)	−0.013 (2)	0.011 (2)	0.004 (2)
C10	0.0153 (17)	0.037 (2)	0.038 (2)	−0.0020 (16)	0.0020 (16)	0.0000 (18)
C11	0.0137 (16)	0.036 (2)	0.035 (2)	−0.0027 (16)	−0.0040 (15)	−0.0016 (17)
C12	0.0119 (15)	0.0186 (17)	0.0336 (19)	−0.0001 (13)	−0.0025 (14)	0.0006 (14)
C13	0.0263 (19)	0.0213 (18)	0.037 (2)	−0.0052 (15)	0.0003 (17)	−0.0005 (16)
C14	0.0166 (16)	0.0253 (19)	0.0312 (19)	−0.0001 (14)	−0.0043 (15)	0.0012 (15)
C15	0.040 (2)	0.031 (2)	0.035 (2)	0.0110 (19)	−0.006 (2)	0.0033 (17)
C16	0.0162 (17)	0.051 (3)	0.035 (2)	0.0011 (18)	−0.0040 (16)	−0.0054 (19)
C17	0.0211 (19)	0.050 (3)	0.036 (2)	0.0044 (18)	−0.0061 (17)	−0.0037 (19)
C18	0.0198 (17)	0.029 (2)	0.031 (2)	−0.0028 (15)	−0.0044 (15)	0.0031 (16)
C19	0.0160 (16)	0.0280 (19)	0.0280 (19)	0.0000 (15)	−0.0019 (14)	0.0019 (15)
C20	0.0164 (17)	0.051 (3)	0.035 (2)	−0.0099 (18)	−0.0019 (16)	0.0046 (19)
C21	0.0147 (16)	0.036 (2)	0.032 (2)	0.0000 (15)	−0.0041 (15)	−0.0007 (17)
C22	0.0162 (16)	0.0172 (17)	0.0312 (19)	−0.0044 (14)	−0.0049 (14)	0.0044 (14)
C23	0.0206 (18)	0.033 (2)	0.0264 (19)	−0.0018 (15)	−0.0035 (15)	0.0044 (16)
C24	0.037 (2)	0.032 (2)	0.039 (2)	−0.0037 (19)	0.0009 (19)	0.0018 (18)
C25	0.0211 (18)	0.040 (2)	0.033 (2)	0.0024 (17)	−0.0060 (16)	0.0050 (17)
C26	0.0167 (18)	0.039 (2)	0.043 (2)	0.0027 (17)	−0.0018 (17)	0.0003 (19)
C27	0.0190 (17)	0.033 (2)	0.032 (2)	0.0029 (16)	0.0004 (15)	0.0013 (16)
C28	0.0219 (18)	0.044 (2)	0.032 (2)	0.0107 (18)	−0.0011 (16)	−0.0043 (19)
C29	0.034 (2)	0.051 (3)	0.030 (2)	0.008 (2)	0.0037 (18)	0.0006 (19)
C30	0.034 (2)	0.054 (3)	0.056 (3)	−0.005 (2)	0.005 (2)	−0.019 (2)
C31	0.027 (2)	0.037 (2)	0.039 (2)	0.0070 (18)	0.0017 (18)	0.0046 (18)
C32	0.0238 (19)	0.032 (2)	0.049 (3)	0.0037 (17)	0.0128 (18)	0.0014 (19)
C33	0.0146 (16)	0.034 (2)	0.031 (2)	−0.0004 (15)	0.0043 (15)	−0.0001 (16)
C34	0.0174 (16)	0.0246 (19)	0.0268 (19)	−0.0001 (14)	0.0018 (14)	0.0032 (15)
C35	0.029 (2)	0.036 (2)	0.031 (2)	−0.0009 (18)	0.0025 (17)	0.0097 (17)
C36	0.0178 (17)	0.037 (2)	0.0257 (18)	0.0026 (16)	0.0007 (14)	0.0024 (16)
C37	0.0167 (18)	0.056 (3)	0.032 (2)	0.0015 (18)	−0.0026 (16)	0.0107 (19)
C38	0.049 (3)	0.071 (3)	0.025 (2)	−0.004 (3)	0.004 (2)	−0.002 (2)
C39	0.045 (3)	0.067 (3)	0.048 (3)	0.005 (3)	−0.013 (2)	0.018 (2)
C40	0.0159 (17)	0.044 (2)	0.040 (2)	0.0071 (17)	−0.0058 (16)	−0.006 (2)
C41	0.0137 (16)	0.047 (2)	0.033 (2)	0.0036 (17)	0.0050 (15)	−0.0090 (19)
C42	0.0117 (15)	0.0279 (19)	0.0305 (19)	0.0025 (14)	0.0003 (14)	−0.0017 (15)
C43	0.036 (2)	0.033 (2)	0.035 (2)	0.0115 (19)	−0.0056 (19)	−0.0004 (17)
C44	0.0163 (16)	0.032 (2)	0.0254 (18)	−0.0035 (15)	0.0037 (14)	0.0011 (15)
C45	0.044 (3)	0.035 (2)	0.034 (2)	−0.019 (2)	0.008 (2)	0.0038 (18)
C46	0.0126 (17)	0.062 (3)	0.036 (2)	0.0050 (18)	0.0047 (16)	−0.003 (2)
C47	0.0208 (19)	0.049 (3)	0.032 (2)	−0.0058 (18)	0.0072 (16)	−0.0024 (18)
C48	0.0176 (17)	0.032 (2)	0.0278 (19)	−0.0023 (15)	0.0043 (15)	0.0022 (16)
C49	0.0169 (16)	0.0241 (18)	0.0277 (18)	0.0015 (15)	0.0026 (14)	0.0027 (15)
C50	0.0155 (16)	0.041 (2)	0.028 (2)	0.0077 (16)	0.0015 (14)	0.0045 (17)
C51	0.0133 (16)	0.036 (2)	0.0268 (19)	−0.0013 (15)	0.0024 (14)	0.0031 (16)
C52	0.0160 (16)	0.0182 (17)	0.0259 (18)	0.0008 (13)	0.0025 (14)	0.0030 (14)
C53	0.0200 (17)	0.031 (2)	0.0242 (18)	0.0007 (15)	0.0039 (15)	0.0031 (15)
C54	0.036 (2)	0.032 (2)	0.041 (2)	0.0001 (18)	−0.0008 (19)	−0.0029 (18)
C55	0.0184 (17)	0.038 (2)	0.033 (2)	0.0011 (16)	0.0062 (16)	0.0020 (17)
C56	0.0168 (17)	0.038 (2)	0.037 (2)	0.0036 (16)	0.0029 (16)	−0.0001 (18)
C57	0.0223 (18)	0.0259 (19)	0.034 (2)	0.0010 (16)	−0.0015 (16)	0.0042 (16)
C58	0.0206 (18)	0.046 (2)	0.032 (2)	−0.0077 (18)	−0.0003 (16)	0.0062 (18)
C59	0.035 (2)	0.036 (2)	0.042 (2)	−0.0033 (19)	−0.0108 (19)	−0.0029 (19)
C60	0.042 (3)	0.054 (3)	0.057 (3)	0.011 (2)	−0.008 (2)	−0.019 (2)

### Geometric parameters (Å, °)

**Table:**

O1—C1	1.304 (5)	C27—C28	1.547 (6)
O1—H1	0.84	C28—C30	1.512 (7)
O2—C1	1.223 (6)	C28—C29	1.511 (6)
O3—C27	1.414 (5)	C29—H29A	0.98
O3—C23	1.470 (5)	C29—H29B	0.98
O4—C27	1.417 (5)	C29—H29C	0.98
O4—H4	0.84	C30—H30A	0.98
O5—C28	1.456 (5)	C30—H30B	0.98
O5—H5	0.84	C30—H30C	0.98
O6—C31	1.308 (5)	C31—C32	1.494 (6)
O6—H6	0.84	C32—C33	1.521 (6)
O7—C31	1.224 (5)	C32—H32A	0.99
O8—C57	1.424 (5)	C32—H32B	0.99
O8—C53	1.460 (5)	C33—C34	1.556 (5)
O9—C57	1.407 (5)	C33—H33A	0.99
O9—H9	0.84	C33—H33B	0.99
O10—C58	1.427 (5)	C34—C35	1.531 (5)
O10—H10	0.84	C34—C36	1.564 (5)
C1—C2	1.496 (5)	C34—C52	1.570 (5)
C2—C3	1.519 (5)	C35—H35A	0.98
C2—H2A	0.99	C35—H35B	0.98
C2—H2B	0.99	C35—H35C	0.98
C3—C4	1.560 (5)	C36—C37	1.529 (5)
C3—H3A	0.99	C36—C40	1.536 (5)
C3—H3B	0.99	C36—H36	1.00
C4—C5	1.542 (5)	C37—C38	1.404 (7)
C4—C6	1.562 (5)	C37—C39	1.424 (6)
C4—C22	1.571 (5)	C38—H38A	0.95
C5—H5A	0.98	C38—H38B	0.95
C5—H5B	0.98	C39—H39A	0.98
C5—H5C	0.98	C39—H39B	0.98
C6—C7	1.523 (5)	C39—H39C	0.98
C6—C10	1.535 (5)	C40—C41	1.526 (6)
C6—H6A	1.00	C40—H40A	0.99
C7—C9	1.392 (6)	C40—H40B	0.99
C7—C8	1.439 (6)	C41—C42	1.531 (5)
C8—H8A	0.98	C41—H41A	0.99
C8—H8B	0.98	C41—H41B	0.99
C8—H8C	0.98	C42—C43	1.526 (5)
C9—H9A	0.95	C42—C52	1.563 (5)
C9—H9B	0.95	C42—C44	1.581 (5)
C10—C11	1.522 (6)	C43—H43A	0.98
C10—H10A	0.99	C43—H43B	0.98
C10—H10B	0.99	C43—H43C	0.98
C11—C12	1.539 (5)	C44—C46	1.542 (5)
C11—H11A	0.99	C44—C49	1.543 (5)
C11—H11B	0.99	C44—C45	1.546 (5)
C12—C13	1.547 (5)	C45—H45A	0.98
C12—C22	1.557 (5)	C45—H45B	0.98
C12—C14	1.570 (5)	C45—H45C	0.98
C13—H13A	0.98	C46—C47	1.539 (6)
C13—H13B	0.98	C46—H46A	0.99
C13—H13C	0.98	C46—H46B	0.99
C14—C19	1.547 (5)	C47—C48	1.566 (5)
C14—C15	1.553 (5)	C47—H48A	0.99
C14—C16	1.551 (5)	C47—H48B	0.99
C15—H15A	0.98	C48—C53	1.533 (5)
C15—H15B	0.98	C48—C49	1.550 (5)
C15—H15C	0.98	C48—H48	1.00
C16—C17	1.540 (6)	C49—C50	1.512 (5)
C16—H16A	0.99	C49—H49	1.00
C16—H16B	0.99	C50—C51	1.537 (5)
C17—C18	1.564 (5)	C50—H50A	0.99
C17—H17A	0.99	C50—H50B	0.99
C17—H17B	0.99	C51—C52	1.538 (5)
C18—C23	1.530 (6)	C51—H51A	0.99
C18—C19	1.539 (5)	C51—H51B	0.99
C18—H18	1.00	C52—H52	1.00
C19—C20	1.522 (5)	C53—C54	1.510 (6)
C19—H19	1.00	C53—C55	1.542 (5)
C20—C21	1.539 (6)	C54—H54A	0.98
C20—H20A	0.99	C54—H54B	0.98
C20—H20B	0.99	C54—H54C	0.98
C21—C22	1.534 (5)	C55—C56	1.522 (6)
C21—H21A	0.99	C55—H55A	0.99
C21—H21B	0.99	C55—H55B	0.99
C22—H22	1.00	C56—C57	1.533 (5)
C23—C24	1.523 (6)	C56—H56A	0.99
C23—C25	1.540 (5)	C56—H56B	0.99
C24—H24A	0.98	C57—C58	1.531 (6)
C24—H24B	0.98	C58—C59	1.509 (6)
C24—H24C	0.98	C58—C60	1.534 (7)
C25—C26	1.511 (6)	C59—H59A	0.98
C25—H25A	0.99	C59—H59B	0.98
C25—H25B	0.99	C59—H59C	0.98
C26—C27	1.526 (5)	C60—H60A	0.98
C26—H26A	0.99	C60—H60B	0.98
C26—H26B	0.99	C60—H60C	0.98
			
C1—O1—H1	109.5	H30A—C30—H30B	109.5
C27—O3—C23	111.0 (3)	C28—C30—H30C	109.5
C27—O4—H4	109.5	H30A—C30—H30C	109.5
C28—O5—H5	109.5	H30B—C30—H30C	109.5
C31—O6—H6	109.5	O7—C31—O6	123.1 (4)
C57—O8—C53	111.4 (3)	O7—C31—C32	123.3 (4)
C57—O9—H9	109.5	O6—C31—C32	113.6 (4)
C58—O10—H10	109.5	C31—C32—C33	112.1 (3)
O2—C1—O1	123.3 (4)	C31—C32—H32A	109.2
O2—C1—C2	122.7 (4)	C33—C32—H32A	109.2
O1—C1—C2	113.8 (4)	C31—C32—H32B	109.2
C1—C2—C3	112.5 (3)	C33—C32—H32B	109.2
C1—C2—H2A	109.1	H32A—C32—H32B	107.9
C3—C2—H2A	109.1	C32—C33—C34	117.4 (3)
C1—C2—H2B	109.1	C32—C33—H33A	107.9
C3—C2—H2B	109.1	C34—C33—H33A	107.9
H2A—C2—H2B	107.8	C32—C33—H33B	107.9
C2—C3—C4	117.3 (3)	C34—C33—H33B	107.9
C2—C3—H3A	108.0	H33A—C33—H33B	107.2
C4—C3—H3A	108.0	C35—C34—C33	104.7 (3)
C2—C3—H3B	108.0	C35—C34—C36	112.7 (3)
C4—C3—H3B	108.0	C33—C34—C36	108.9 (3)
H3A—C3—H3B	107.2	C35—C34—C52	113.4 (3)
C5—C4—C3	105.1 (3)	C33—C34—C52	109.3 (3)
C5—C4—C6	112.4 (3)	C36—C34—C52	107.6 (3)
C3—C4—C6	109.2 (3)	C34—C35—H35A	109.5
C5—C4—C22	113.3 (3)	C34—C35—H35B	109.5
C3—C4—C22	109.0 (3)	H35A—C35—H35B	109.5
C6—C4—C22	107.7 (3)	C34—C35—H35C	109.5
C4—C5—H5A	109.5	H35A—C35—H35C	109.5
C4—C5—H5B	109.5	H35B—C35—H35C	109.5
H5A—C5—H5B	109.5	C37—C36—C40	110.6 (3)
C4—C5—H5C	109.5	C37—C36—C34	115.9 (3)
H5A—C5—H5C	109.5	C40—C36—C34	112.3 (3)
H5B—C5—H5C	109.5	C37—C36—H36	105.8
C7—C6—C10	110.7 (3)	C40—C36—H36	105.8
C7—C6—C4	115.8 (3)	C34—C36—H36	105.8
C10—C6—C4	111.7 (3)	C38—C37—C39	119.3 (4)
C7—C6—H6A	106.0	C38—C37—C36	118.5 (4)
C10—C6—H6A	106.0	C39—C37—C36	122.0 (4)
C4—C6—H6A	106.0	C37—C38—H38A	120.0
C9—C7—C8	119.5 (4)	C37—C38—H38B	120.0
C9—C7—C6	122.5 (4)	H38A—C38—H38B	120.0
C8—C7—C6	117.9 (4)	C37—C39—H39A	109.5
C7—C8—H8A	109.5	C37—C39—H39B	109.5
C7—C8—H8B	109.5	H39A—C39—H39B	109.5
H8A—C8—H8B	109.5	C37—C39—H39C	109.5
C7—C8—H8C	109.5	H39A—C39—H39C	109.5
H8A—C8—H8C	109.5	H39B—C39—H39C	109.5
H8B—C8—H8C	109.5	C41—C40—C36	110.9 (3)
C7—C9—H9A	120.0	C41—C40—H40A	109.5
C7—C9—H9B	120.0	C36—C40—H40A	109.5
H9A—C9—H9B	120.0	C41—C40—H40B	109.5
C11—C10—C6	111.9 (3)	C36—C40—H40B	109.5
C11—C10—H10A	109.2	H40A—C40—H40B	108.0
C6—C10—H10A	109.2	C40—C41—C42	113.3 (3)
C11—C10—H10B	109.2	C40—C41—H41A	108.9
C6—C10—H10B	109.2	C42—C41—H41A	108.9
H10A—C10—H10B	107.9	C40—C41—H41B	108.9
C10—C11—C12	113.5 (3)	C42—C41—H41B	108.9
C10—C11—H11A	108.9	H41A—C41—H41B	107.7
C12—C11—H11A	108.9	C43—C42—C41	107.8 (3)
C10—C11—H11B	108.9	C43—C42—C52	112.4 (3)
C12—C11—H11B	108.9	C41—C42—C52	108.9 (3)
H11A—C11—H11B	107.7	C43—C42—C44	110.5 (3)
C11—C12—C13	108.0 (3)	C41—C42—C44	110.9 (3)
C11—C12—C22	109.1 (3)	C52—C42—C44	106.4 (3)
C13—C12—C22	112.1 (3)	C42—C43—H43A	109.5
C11—C12—C14	110.7 (3)	C42—C43—H43B	109.5
C13—C12—C14	109.9 (3)	H43A—C43—H43B	109.5
C22—C12—C14	107.1 (3)	C42—C43—H43C	109.5
C12—C13—H13A	109.5	H43A—C43—H43C	109.5
C12—C13—H13B	109.5	H43B—C43—H43C	109.5
H13A—C13—H13B	109.5	C46—C44—C49	100.0 (3)
C12—C13—H13C	109.5	C46—C44—C45	106.4 (3)
H13A—C13—H13C	109.5	C49—C44—C45	110.9 (3)
H13B—C13—H13C	109.5	C46—C44—C42	116.5 (3)
C19—C14—C15	110.5 (3)	C49—C44—C42	109.7 (3)
C19—C14—C16	100.1 (3)	C45—C44—C42	112.5 (3)
C15—C14—C16	105.4 (3)	C44—C45—H45A	109.5
C19—C14—C12	109.9 (3)	C44—C45—H45B	109.5
C15—C14—C12	112.5 (3)	H45A—C45—H45B	109.5
C16—C14—C12	117.7 (3)	C44—C45—H45C	109.5
C14—C15—H15A	109.5	H45A—C45—H45C	109.5
C14—C15—H15B	109.5	H45B—C45—H45C	109.5
H15A—C15—H15B	109.5	C47—C46—C44	104.0 (3)
C14—C15—H15C	109.5	C47—C46—H46A	110.9
H15A—C15—H15C	109.5	C44—C46—H46A	110.9
H15B—C15—H15C	109.5	C47—C46—H46B	110.9
C17—C16—C14	104.3 (3)	C44—C46—H46B	110.9
C17—C16—H16A	110.9	H46A—C46—H46B	109.0
C14—C16—H16A	110.9	C46—C47—C48	106.1 (3)
C17—C16—H16B	110.9	C46—C47—H48A	110.5
C14—C16—H16B	110.9	C48—C47—H48A	110.5
H16A—C16—H16B	108.9	C46—C47—H48B	110.5
C16—C17—C18	106.1 (3)	C48—C47—H48B	110.5
C16—C17—H17A	110.5	H48A—C47—H48B	108.7
C18—C17—H17A	110.5	C53—C48—C49	115.0 (3)
C16—C17—H17B	110.5	C53—C48—C47	112.9 (3)
C18—C17—H17B	110.5	C49—C48—C47	103.8 (3)
H17A—C17—H17B	108.7	C53—C48—H48	108.3
C23—C18—C19	115.5 (3)	C49—C48—H48	108.3
C23—C18—C17	113.0 (3)	C47—C48—H48	108.3
C19—C18—C17	104.4 (3)	C50—C49—C44	111.8 (3)
C23—C18—H18	107.9	C50—C49—C48	119.7 (3)
C19—C18—H18	107.9	C44—C49—C48	105.3 (3)
C17—C18—H18	107.9	C50—C49—H49	106.4
C20—C19—C18	119.6 (3)	C44—C49—H49	106.4
C20—C19—C14	110.8 (3)	C48—C49—H49	106.4
C18—C19—C14	105.2 (3)	C49—C50—C51	109.6 (3)
C20—C19—H19	106.8	C49—C50—H50A	109.7
C18—C19—H19	106.8	C51—C50—H50A	109.7
C14—C19—H19	106.8	C49—C50—H50B	109.7
C19—C20—C21	110.7 (3)	C51—C50—H50B	109.7
C19—C20—H20A	109.5	H50A—C50—H50B	108.2
C21—C20—H20A	109.5	C52—C51—C50	112.9 (3)
C19—C20—H20B	109.5	C52—C51—H51A	109.0
C21—C20—H20B	109.5	C50—C51—H51A	109.0
H20A—C20—H20B	108.1	C52—C51—H51B	109.0
C22—C21—C20	113.4 (3)	C50—C51—H51B	109.0
C22—C21—H21A	108.9	H51A—C51—H51B	107.8
C20—C21—H21A	108.9	C51—C52—C42	112.0 (3)
C22—C21—H21B	108.9	C51—C52—C34	113.3 (3)
C20—C21—H21B	108.9	C42—C52—C34	117.3 (3)
H21A—C21—H21B	107.7	C51—C52—H52	104.2
C21—C22—C12	111.9 (3)	C42—C52—H52	104.2
C21—C22—C4	113.4 (3)	C34—C52—H52	104.2
C12—C22—C4	117.1 (3)	O8—C53—C54	107.1 (3)
C21—C22—H22	104.3	O8—C53—C48	106.6 (3)
C12—C22—H22	104.3	C54—C53—C48	113.5 (3)
C4—C22—H22	104.3	O8—C53—C55	103.8 (3)
O3—C23—C24	106.8 (3)	C54—C53—C55	111.3 (3)
O3—C23—C18	106.6 (3)	C48—C53—C55	113.7 (3)
C24—C23—C18	113.7 (3)	C53—C54—H54A	109.5
O3—C23—C25	103.7 (3)	C53—C54—H54B	109.5
C24—C23—C25	111.1 (4)	H54A—C54—H54B	109.5
C18—C23—C25	114.0 (3)	C53—C54—H54C	109.5
C23—C24—H24A	109.5	H54A—C54—H54C	109.5
C23—C24—H24B	109.5	H54B—C54—H54C	109.5
H24A—C24—H24B	109.5	C56—C55—C53	103.4 (3)
C23—C24—H24C	109.5	C56—C55—H55A	111.1
H24A—C24—H24C	109.5	C53—C55—H55A	111.1
H24B—C24—H24C	109.5	C56—C55—H55B	111.1
C26—C25—C23	103.5 (3)	C53—C55—H55B	111.1
C26—C25—H25A	111.1	H55A—C55—H55B	109.0
C23—C25—H25A	111.1	C55—C56—C57	101.0 (3)
C26—C25—H25B	111.1	C55—C56—H56A	111.6
C23—C25—H25B	111.1	C57—C56—H56A	111.6
H25A—C25—H25B	109.0	C55—C56—H56B	111.6
C25—C26—C27	101.5 (3)	C57—C56—H56B	111.6
C25—C26—H26A	111.5	H56A—C56—H56B	109.4
C27—C26—H26A	111.5	O9—C57—O8	110.6 (3)
C25—C26—H26B	111.5	O9—C57—C58	109.9 (3)
C27—C26—H26B	111.5	O8—C57—C58	108.1 (3)
H26A—C26—H26B	109.3	O9—C57—C56	107.3 (3)
O4—C27—O3	110.0 (3)	O8—C57—C56	104.8 (3)
O4—C27—C26	106.8 (3)	C58—C57—C56	116.0 (3)
O3—C27—C26	105.5 (3)	O10—C58—C59	111.3 (3)
O4—C27—C28	109.2 (3)	O10—C58—C60	105.9 (4)
O3—C27—C28	108.9 (3)	C59—C58—C60	110.3 (4)
C26—C27—C28	116.2 (3)	O10—C58—C57	105.9 (3)
O5—C28—C30	105.6 (4)	C59—C58—C57	111.3 (4)
O5—C28—C29	108.5 (3)	C60—C58—C57	111.9 (4)
C30—C28—C29	112.4 (4)	C58—C59—H59A	109.5
O5—C28—C27	107.4 (3)	C58—C59—H59B	109.5
C30—C28—C27	112.0 (4)	H59A—C59—H59B	109.5
C29—C28—C27	110.6 (3)	C58—C59—H59C	109.5
C28—C29—H29A	109.5	H59A—C59—H59C	109.5
C28—C29—H29B	109.5	H59B—C59—H59C	109.5
H29A—C29—H29B	109.5	C58—C60—H60A	109.5
C28—C29—H29C	109.5	C58—C60—H60B	109.5
H29A—C29—H29C	109.5	H60A—C60—H60B	109.5
H29B—C29—H29C	109.5	C58—C60—H60C	109.5
C28—C30—H30A	109.5	H60A—C60—H60C	109.5
C28—C30—H30B	109.5	H60B—C60—H60C	109.5
			
O2—C1—C2—C3	38.6 (7)	O7—C31—C32—C33	43.5 (6)
O1—C1—C2—C3	−145.4 (4)	O6—C31—C32—C33	−138.8 (4)
C1—C2—C3—C4	−169.3 (4)	C31—C32—C33—C34	−164.4 (4)
C2—C3—C4—C5	173.1 (4)	C32—C33—C34—C35	172.0 (4)
C2—C3—C4—C6	−66.1 (4)	C32—C33—C34—C36	−67.2 (4)
C2—C3—C4—C22	51.3 (4)	C32—C33—C34—C52	50.1 (4)
C5—C4—C6—C7	56.2 (4)	C35—C34—C36—C37	55.7 (4)
C3—C4—C6—C7	−60.1 (4)	C33—C34—C36—C37	−60.1 (4)
C22—C4—C6—C7	−178.3 (3)	C52—C34—C36—C37	−178.5 (3)
C5—C4—C6—C10	−71.7 (4)	C35—C34—C36—C40	−72.7 (4)
C3—C4—C6—C10	172.1 (3)	C33—C34—C36—C40	171.6 (3)
C22—C4—C6—C10	53.8 (4)	C52—C34—C36—C40	53.1 (4)
C10—C6—C7—C9	49.1 (5)	C40—C36—C37—C38	−122.2 (4)
C4—C6—C7—C9	−79.2 (5)	C34—C36—C37—C38	108.6 (4)
C10—C6—C7—C8	−126.1 (4)	C40—C36—C37—C39	53.7 (5)
C4—C6—C7—C8	105.5 (4)	C34—C36—C37—C39	−75.5 (5)
C7—C6—C10—C11	170.8 (3)	C37—C36—C40—C41	169.7 (4)
C4—C6—C10—C11	−58.7 (4)	C34—C36—C40—C41	−59.2 (4)
C6—C10—C11—C12	57.0 (5)	C36—C40—C41—C42	58.8 (5)
C10—C11—C12—C13	71.5 (4)	C40—C41—C42—C43	69.9 (4)
C10—C11—C12—C22	−50.6 (4)	C40—C41—C42—C52	−52.3 (4)
C10—C11—C12—C14	−168.2 (3)	C40—C41—C42—C44	−169.0 (3)
C11—C12—C14—C19	179.8 (3)	C43—C42—C44—C46	50.1 (4)
C13—C12—C14—C19	−61.1 (4)	C41—C42—C44—C46	−69.4 (4)
C22—C12—C14—C19	61.0 (4)	C52—C42—C44—C46	172.3 (3)
C11—C12—C14—C15	56.2 (4)	C43—C42—C44—C49	−62.5 (4)
C13—C12—C14—C15	175.4 (3)	C41—C42—C44—C49	177.9 (3)
C22—C12—C14—C15	−62.6 (4)	C52—C42—C44—C49	59.7 (4)
C11—C12—C14—C16	−66.6 (4)	C43—C42—C44—C45	173.4 (3)
C13—C12—C14—C16	52.6 (4)	C41—C42—C44—C45	53.9 (4)
C22—C12—C14—C16	174.6 (3)	C52—C42—C44—C45	−64.3 (4)
C19—C14—C16—C17	−41.8 (4)	C49—C44—C46—C47	−43.5 (4)
C15—C14—C16—C17	73.0 (4)	C45—C44—C46—C47	72.0 (4)
C12—C14—C16—C17	−160.7 (3)	C42—C44—C46—C47	−161.6 (3)
C14—C16—C17—C18	25.7 (4)	C44—C46—C47—C48	28.2 (4)
C16—C17—C18—C23	127.3 (4)	C46—C47—C48—C53	123.9 (4)
C16—C17—C18—C19	1.1 (5)	C46—C47—C48—C49	−1.3 (4)
C23—C18—C19—C20	82.3 (4)	C46—C44—C49—C50	174.8 (3)
C17—C18—C19—C20	−152.9 (4)	C45—C44—C49—C50	62.8 (4)
C23—C18—C19—C14	−152.3 (3)	C42—C44—C49—C50	−62.2 (4)
C17—C18—C19—C14	−27.6 (4)	C46—C44—C49—C48	43.2 (4)
C15—C14—C19—C20	62.8 (4)	C45—C44—C49—C48	−68.8 (4)
C16—C14—C19—C20	173.6 (3)	C42—C44—C49—C48	166.2 (3)
C12—C14—C19—C20	−61.9 (4)	C53—C48—C49—C50	83.0 (4)
C15—C14—C19—C18	−67.8 (4)	C47—C48—C49—C50	−153.1 (4)
C16—C14—C19—C18	43.0 (4)	C53—C48—C49—C44	−150.1 (3)
C12—C14—C19—C18	167.5 (3)	C47—C48—C49—C44	−26.2 (4)
C18—C19—C20—C21	178.1 (3)	C44—C49—C50—C51	57.0 (4)
C14—C19—C20—C21	55.5 (4)	C48—C49—C50—C51	−179.2 (3)
C19—C20—C21—C22	−51.7 (5)	C49—C50—C51—C52	−53.5 (4)
C20—C21—C22—C12	53.7 (4)	C50—C51—C52—C42	55.6 (4)
C20—C21—C22—C4	−171.3 (3)	C50—C51—C52—C34	−169.0 (3)
C11—C12—C22—C21	−176.7 (3)	C43—C42—C52—C51	64.1 (4)
C13—C12—C22—C21	63.7 (4)	C41—C42—C52—C51	−176.5 (3)
C14—C12—C22—C21	−56.9 (4)	C44—C42—C52—C51	−56.9 (4)
C11—C12—C22—C4	50.0 (4)	C43—C42—C52—C34	−69.4 (4)
C13—C12—C22—C4	−69.6 (4)	C41—C42—C52—C34	50.0 (4)
C14—C12—C22—C4	169.8 (3)	C44—C42—C52—C34	169.6 (3)
C5—C4—C22—C21	−59.4 (4)	C35—C34—C52—C51	−57.8 (4)
C3—C4—C22—C21	57.3 (4)	C33—C34—C52—C51	58.7 (4)
C6—C4—C22—C21	175.7 (3)	C36—C34—C52—C51	176.8 (3)
C5—C4—C22—C12	73.2 (4)	C35—C34—C52—C42	75.1 (4)
C3—C4—C22—C12	−170.1 (3)	C33—C34—C52—C42	−168.5 (3)
C6—C4—C22—C12	−51.7 (4)	C36—C34—C52—C42	−50.3 (4)
C27—O3—C23—C24	−112.4 (4)	C57—O8—C53—C54	−113.8 (3)
C27—O3—C23—C18	125.8 (3)	C57—O8—C53—C48	124.4 (3)
C27—O3—C23—C25	5.1 (4)	C57—O8—C53—C55	4.0 (4)
C19—C18—C23—O3	176.0 (3)	C49—C48—C53—O8	175.9 (3)
C17—C18—C23—O3	55.9 (4)	C47—C48—C53—O8	57.1 (4)
C19—C18—C23—C24	58.7 (4)	C49—C48—C53—C54	58.3 (4)
C17—C18—C23—C24	−61.5 (5)	C47—C48—C53—C54	−60.6 (4)
C19—C18—C23—C25	−70.2 (4)	C49—C48—C53—C55	−70.2 (4)
C17—C18—C23—C25	169.7 (4)	C47—C48—C53—C55	170.9 (3)
O3—C23—C25—C26	−27.0 (4)	O8—C53—C55—C56	−26.8 (4)
C24—C23—C25—C26	87.4 (4)	C54—C53—C55—C56	88.1 (4)
C18—C23—C25—C26	−142.5 (3)	C48—C53—C55—C56	−142.3 (3)
C23—C25—C26—C27	37.5 (4)	C53—C55—C56—C57	38.0 (4)
C23—O3—C27—O4	−96.1 (3)	C53—O8—C57—O9	−95.0 (4)
C23—O3—C27—C26	18.8 (4)	C53—O8—C57—C58	144.7 (3)
C23—O3—C27—C28	144.2 (3)	C53—O8—C57—C56	20.3 (4)
C25—C26—C27—O4	82.0 (4)	C55—C56—C57—O9	81.5 (4)
C25—C26—C27—O3	−35.0 (4)	C55—C56—C57—O8	−36.1 (4)
C25—C26—C27—C28	−155.8 (4)	C55—C56—C57—C58	−155.2 (3)
O4—C27—C28—O5	−51.0 (4)	O9—C57—C58—O10	−61.4 (4)
O3—C27—C28—O5	69.3 (4)	O8—C57—C58—O10	59.5 (4)
C26—C27—C28—O5	−171.8 (3)	C56—C57—C58—O10	176.8 (3)
O4—C27—C28—C30	−166.5 (3)	O9—C57—C58—C59	59.7 (4)
O3—C27—C28—C30	−46.2 (4)	O8—C57—C58—C59	−179.5 (3)
C26—C27—C28—C30	72.7 (5)	C56—C57—C58—C59	−62.1 (5)
O4—C27—C28—C29	67.3 (4)	O9—C57—C58—C60	−176.3 (4)
O3—C27—C28—C29	−172.5 (3)	O8—C57—C58—C60	−55.5 (4)
C26—C27—C28—C29	−53.5 (5)	C56—C57—C58—C60	61.8 (5)

### Hydrogen-bond geometry (Å, °)

**Table:**

*D*—H···*A*	*D*—H	H···*A*	*D*···*A*	*D*—H···*A*
O1—H1···O10	0.84	1.91	2.709 (4)	158
O4—H4···O7^i^	0.84	1.96	2.770 (4)	160
O5—H5···O7^i^	0.84	2.45	3.205 (5)	149
O6—H6···O5^ii^	0.84	1.99	2.780 (5)	157
O9—H9···O2	0.84	1.98	2.807 (5)	171
O10—H10···O5^iii^	0.84	2.15	2.829 (4)	138

Symmetry codes: (i) *x*−1, *y*, *z*; (ii) *x*+1, *y*, *z*; (iii) −*x*+3/2, −*y*+1, *z*+1/2.
